# The Effect of Tacrolimus and Mycophenolic Acid on CD14+ Monocyte Activation and Function

**DOI:** 10.1371/journal.pone.0170806

**Published:** 2017-01-25

**Authors:** Nynke M. Kannegieter, Dennis A. Hesselink, Marjolein Dieterich, Rens Kraaijeveld, Ajda T. Rowshani, Pieter J. M. Leenen, Carla C. Baan

**Affiliations:** 1 Department of Internal Medicine, Section of Transplantation and Nephrology, Erasmus MC, University Medical Center Rotterdam, Rotterdam, The Netherlands; 2 Department of Immunology, Erasmus MC, University Medical Center Rotterdam, Rotterdam, The Netherlands; The University of Hong Kong, HONG KONG

## Abstract

Monocytes and macrophages play key roles in many disease states, including cellular and humoral rejection after solid organ transplantation (SOT). To suppress alloimmunity after SOT, immunosuppressive drug therapy is necessary. However, little is known about the effects of the immunosuppressive drugs tacrolimus and mycophenolic acid (MPA) on monocyte activation and function. Here, the effect of these immunosuppressants on monocytes was investigated by measuring phosphorylation of three intracellular signaling proteins which all have a major role in monocyte function: p38MAPK, ERK and Akt. In addition, biological functions downstream of these signaling pathways were studied, including cytokine production, phagocytosis and differentiation into macrophages. To this end, blood samples from healthy volunteers were spiked with diverse concentrations of tacrolimus and MPA. Tacrolimus (200 ng/ml) inhibited phosphorylation of p38MAPK by 30% (mean) in CD14+ monocytes which was significantly less than in activated CD3+ T cells (max 60%; p < 0.05). This immunosuppressive agent also partly inhibited p-AKT (14%). MPA, at a therapeutic concentration showed the strongest effect on p-AKT (27% inhibition). p-ERK was inhibited with a maximum of 15% after spiking with either tacrolimus or MPA. The production of IL-1β and phagocytosis by monocytes were not affected by tacrolimus concentrations, whereas MPA did inhibit IL-1β production by 50%. Monocyte/macrophage polarization was shifted to an M2-like phenotype in the presence of tacrolimus, while MPA increased the expression of M2 surface markers, including CD163 and CD200R, on M1 macrophages. These results show that tacrolimus and MPA do not strongly affect monocyte function, apart from a change in macrophage polarization, to a clinically relevant degree.

## Introduction

Monocytes have numerous biologic functions that make them key players in solid organ transplantation (SOT)-related conditions, including ischemia-reperfusion injury and its repair, as well as regulation of allograft rejection [1–5]. After SOT, cells of the monocyte/macrophage lineage, process and present alloantigen to the recipients’ immune system, induce inflammation and contribute to allograft rejection, through the secretion of pro-inflammatory cytokines and by providing help to alloreactive T- and B-cells. For example, after ischemia and reperfusion injury, monocytes infiltrate the allograft where, after differentiation into macrophages, they produce inflammatory cytokines, can present donor antigen and also contribute to tissue injury and repair processes [6]. Furthermore, immunohistochemistry of acute rejection kidney specimens demonstrated massive infiltration of the transplanted organ by CD68+ monocyte/macrophages [3, 7]. In antibody-mediated rejection, monocytes control and induce cell injury via the activation of their Fcγ-receptor by allo-antibodies [8, 9]

In tissue, monocytes differentiate into different macrophages subsets depending on the environmental cues the cells encounter. In general, classically and alternatively activated macrophages, termed M1 and M2 macrophages, respectively, represent the ends of a spectrum and can be distinguished by a unique set of cell surface markers [10–12]. Typically, M1 macrophages have a pro-inflammatory function and secrete large amounts of IL-12 and low levels of IL-10, while M2 macrophages can be divided into functionally different subsets. M2a macrophages are involved in T-helper type 2 (Th2) immune responses and have pro-fibrotic properties. M2b macrophages are considered immunoregulatory because they secrete large amounts of IL-10 in combination with TNF-α, IL-1 and IL-6 [13]. Just like the M2a cells, M2b macrophages are also involved in Th2 immune responses [14]. M2c macrophages are anti-inflammatory and characterized by their capacity to produce large amounts of TGF-ß and IL-10 [14, 15].

Despite their clinical importance, surprisingly little is known about the effects of immunosuppressive drugs on monocyte/macrophage differentiation and function. Currently, most kidney transplant recipients, as well as the majority of recipients of other solid organ transplants receive combination immunosuppressive therapy consisting of tacrolimus and mycophenolic acid (MPA; either in the form of mycophenolate mofetil or mycophenolate-sodium) [16–24]. In addition, tacrolimus has also shown to be effective in the treatment of patients with ulcerative colitis and atopic dermatitis [25–28] while MPA is used for the treatment of auto-immune disease such as systemic lupus erythematosus [29]. The limited number of studies on the effect of tacrolimus on monocyte functions have been mostly performed in animal models, immortalized cell lines and cord blood cells. These studies report that tacrolimus can suppress the production of IL-1β, IL-10 and TNF-α by polyclonally activated monocytes [26, 30, 31].

A limited number of studies report on the effects of MPA on macrophage functions. The study by Weimer et al. showed that MPA can suppress the production of IL-1β and IL-6 by staphylococcal superantigens activated monocytes, while the effects of MPA on phagocytosis and monocyte differentiation are unknown [32].

**Fig 1:**
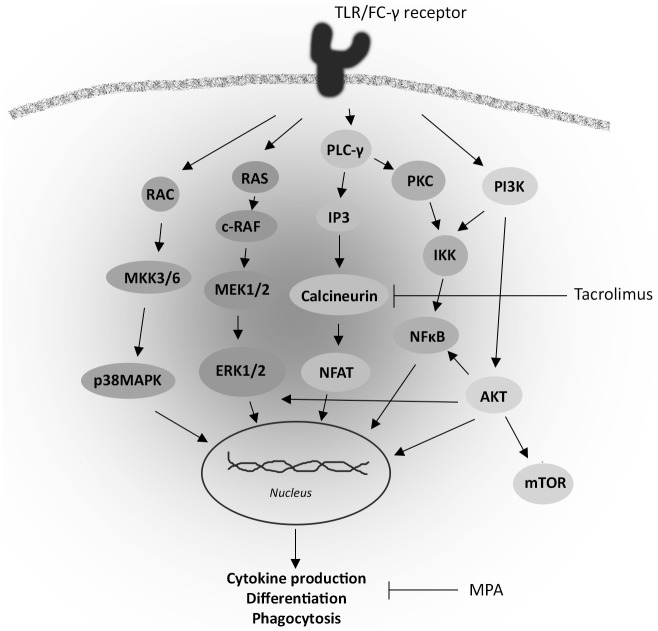
Simplified overview of intracellular signaling pathways involved in monocyte activation. After toll like receptor (TLR) or FC-γ receptor activation the MAPK, Akt and NFAT pathways are activated upstream. The phosphorylation of the intracellular signaling proteins leads to the activation of transcription factors, such as CREB and NF-κB p65, which after activation leads to gene transcription. This process determines the function of monocytes including phenotypic differentiation, cytokine production and phagocytosis.

Monocyte/macrophage responses to environmental triggers are controlled by the activation of multiple intracellular signaling pathways, in which p38 mitogen-activated protein kinases (p38MAPK), extracellular signal-regulated kinases (ERK) and Akt play important roles (Fig 1) [33–36]. Activation of these pathways initiates a complex cascade leading to binding of transcription factors to DNA followed by cytokine gene expression and production, phagocytosis and other functions. Previous studies in T-lymphocytes have demonstrated that tacrolimus does not only inhibit the calcineurin pathway, but also affects the MAPK pathway, while the effects of MPA are unknown [37]. It is unknown if immunosuppressive drugs also inhibit these same pathways in monocytes, and affect related biological functions.

Given the important role of monocytes in immune responses after SOT, the effects on monocyte function of the two most commonly prescribed immunosuppressive agents, tacrolimus and MPA, have been studied. Monocytes have been studied in peripheral blood samples of healthy volunteers that were incubated with tacrolimus or MPA and subsequently their activation, signal transduction, phenotypic differentiation, phagocytic capacity and cytokine production upon PMA/ionomycin stimulation were investigated.

## Materials and Methods

### In vitro phosphorylation study and whole-blood phospho-specific flowcytometry

To measure the effect of tacrolimus and MPA on signaling molecules, heparinized blood samples were drawn from healthy volunteers (n = 5). The study protocol was approved by the local ethics committee of the Erasmus medical center and written informed consent was obtained from each individual after receiving detailed information about the aims of the study. Samples were incubated for one hour at 37°C with either vehicle, tacrolimus (10, 50 or 200 ng/ml; Prograf^®^, Astellas Pharma Inc., Tokyo, Japan), MPA (10 μg/ml; Sigma-Aldrich, Steinheim, Germany) or the p38MAPK inhibitor SB203580 (20 μM; Invivogen, San Diego, CA). The vehicle used was IMDM medium (Gibco BRL, Carlsbad, CA) supplemented with 10% heat-inactivated fetal bovine serum (BioWhittaker, Verviers, Belgium). Phosphorylation of p38MAPK, ERK and Akt was measured according to the manufacturer’s instructions for phosphoprotein analysis (BD Biosciences, San Jose, CA). In brief, 200 μl heparinized blood was stained with Brilliant Violet (BV)510-labelled mouse anti-human CD3 (Biolegend, San Diego, CA) and Fluorescein Isothiocyanate (FITC)-labelled mouse anti-human CD14 (Serotec, Oxford, UK) for 30 minutes at 37°C. After 15 minutes, blood samples were activated with PMA/ionomycin (25 μg per ml/500 μg per ml for p38MAPK and Akt; 5 μg per ml/100 μg per ml for ERK, Sigma-Aldrich, Steinheim, Germany) for 15 minutes. To compare phosphorylation of p38MAPK in CD3+ T-cells and CD14+ monocytes, samples were also stimulated for 30 minutes with PMA/ionomycin. Then cells were fixed for 10 minutes with Lyse/Fix buffer (BD Biosciences) and permeabilized with 90% methanol at -20°C for 30 minutes. Samples were stained intracellular with fluorchrome conjugated mAb phycoerythrin (PE)-labelled mouse anti-p38MAPK (clone pT180/pY182), PE-labelled mouse anti-pAkt (clone pS473) or AlexaFluor647 (AF647)-labelled mouse anti-pERK1/2 (pT202/pY204) (all from BD Biosciences) for 30 minutes at room temperature and analyzed on a FACS Canto II flowcytometer (BD Biosciences). Unstimulated samples were used as negative controls. Isotype controls; mouse IgG1-PE (p38, Akt Biolegend) and mouse IgG1-AF647 (ERK; Biolegend); were included in separated tubes. Interday-variability of the flowcytometer was corrected by using Cytocalbeads (Thermo Scientific, Fremont, CA) according to the manufacturer’s instructions.

### Cytokine production

Heparinized blood samples from healthy volunteers were incubated for 1 hour at 37°C with either vehicle, tacrolimus (10 ng/ml and 50 ng/ml), MPA (10 μg/ml) and activated with PMA/ionomycin (25 μg per ml/400 μg per ml) for four hours at 37°C. Golgiplug (BD Biosciences) was added during the incubation phase to accumulate cytokines intracellularly. Subsequently, EDTA was added for 15 minutes to stop activation. Cells were then stained with BV510-labelled mouse anti-human CD3 (Biolegend) and FITC-labelled mouse anti-human CD14 (Serotec) for 30 minutes at 37°C, fixed for 10 minutes with FACS lysing solution (BD Biosciences) and treated with permeabilization buffer II (BD Biosciences) for 10 min. AF647-labelled anti-IL-1β (detecting the bioactive form of IL-1β, 17.3 kD, clone JK1B-1, BD Biosciences) was used for intracellular cytokine staining for 30 minutes at 4°C.

### Phagocytosis

To assess phagocytosis by monocytes, whole-blood samples of healthy volunteers were incubated for 1 hour at 37°C with either vehicle, tacrolimus (10, 50 and 200 ng/ml) or MPA (10 μg/ml). Then 100 μl of spiked blood per sample was tested for phagocytosis according to the manufacturer’s instructions of the Phagotest (Glycotope Biotechnology, Heidelberg, Germany), which used FITC- labelled E. coli-bacteria.

### Macrophage phenotypic differentiation

Peripheral blood mononuclear cells (PBMC) were isolated from heparinized blood samples by density-gradient centrifugation using Ficoll-paque (GE Healthcare, Uppsala, Sweden). Subsequently, monocytes were isolated by MACS magnetic cell separation (Miltenyi Biotec, Bergisch Gladbach, Germany) using anti-human CD14 magnetic microbeads (Miltenyi). Then, monocytes were cultured in a 12 wells plate at a concentration of 75 x10^4^ cells/ml in RPMI 1640 culture medium with glutaMAX and 25 mM HEPES (Gibco, Life technologies, Paisley, UK), supplemented with 10% heat inactivated fetal bovine serum (FBS, BioWhittaker, Verviers, Belgium) and M-CSF (macrophage colony-stimulating factor; 5ng/ml, Bioconnect, Huissen, the Netherlands). As positive controls, cells were polarized with recombinant human IFN-γ, IL-10 and IL-4 (all 50 ng/ml, Bioconnect) according to Ambarus et al. [38]. Effects of immunosuppressive drugs on monocyte differentiation were examined by culturing monocytes in the presence of either vehicle, tacrolimus (10 ng/ml and 50 ng/ml) or MPA (10 μg/ml). At baseline (day 0) and after 4 days of differentiation, monocytes were tested for their polarization profile after incubation on ice for 1 h by using the following markers [38]: BV421-labelled mouse anti-human CD80 (Biolegend), FITC-labelled mouse anti-human CD163 (Serotec), allophycocyanin (APC)-H7-labelled mouse anti-human CD14 (BD Biosciences), BV510-labelled mouse anti-human CD64 (BD Biosciences), PE-Cyanine7 (PE-Cy7)-labelled mouse anti-human CD16 (BD Biosciences), peridinin-chlorophyll-protein (PERCP)-labelled mouse anti-human CD200R (eBioscience, Vienna, Austria) and APC-labelled rat anti-mouse/human CD11b (Biolegend). Unstained samples were used as a negative control. Background fluorescence levels of isotype controls were used as negative reference.

### Data analysis and statistics

The phosphorylation of p38MAPK, Akt and ERK was measured as the Median Fluorescence Intensity (MFI) and normalized using Cytocalbeads (Thermo Scientific). MFI values of the unstimulated samples were subtracted from the stimulated MFI values. Data and statistical analysis was performed with Diva-version 6.0 software (BD Biosciences) and Graph Pad Prism 5.0 (Graph Pad Software Inc., La Jolla, CA) by using paired t-tests (for the *in vitro* phosphorylation study after performing log transformation and after finding a p-value >0.05 with an F-test). A two-sided p-value < 0.05 was considered statistically significant.

## Results

### Inhibitory effect of tacrolimus and MPA in monocyte signaling pathway activation

**Fig 2:**
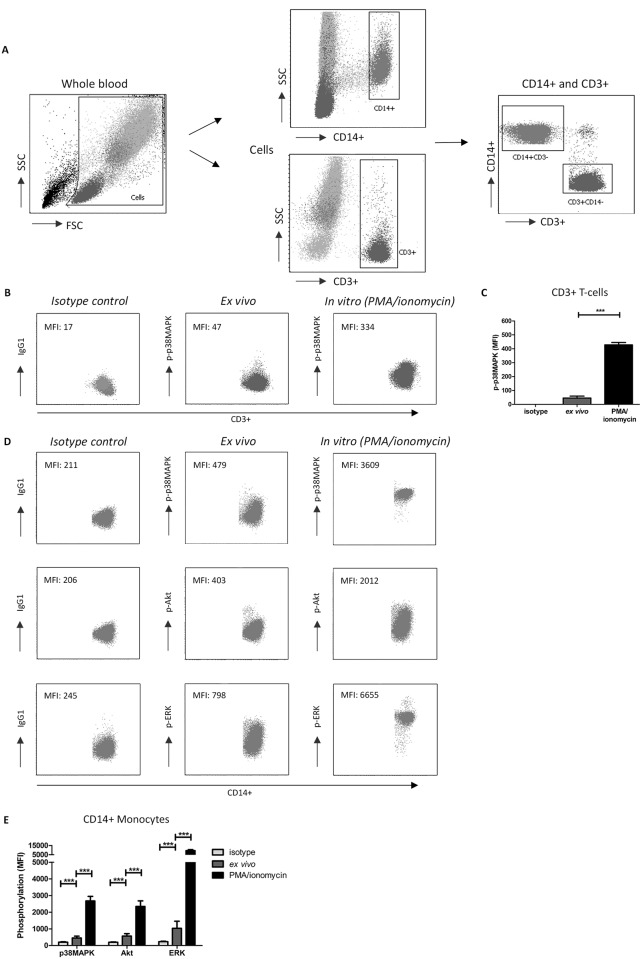
Gating strategy for the selection of monocytes and T-cells, and the measurement of p38MAPK, ERK and Akt phosphorylation. (A) Scatter dot plots to define the monocyte population in blood samples from healthy controls. Cells were selected in whole blood samples for each healthy control and then gated for their expression of either CD3 or CD14. Then, CD3+ and CD14+ cells were combined in one dotplot, to make sure that there were no double positive cells in the analysis. (B) An example of p38MAPK phosphorylation in CD3+ T cells, measured as the median fluorescence intensity (MFI) prior to (ex vivo) or after stimulation with PMA/ionomycin (in vitro) compared to the isotype control. (C) p38MAPK phosphorylation in CD3+ T-cells was increased after stimulation with PMA/ionomycin compared to isotype controls and ex vivo (unstimulated) samples. (D) Examples of the phosphorylation of p38MAPK, Akt and ERK in CD14+ monocytes of isotype controls, ex vivo (unstimulated) and PMA/Ionomycin stimulated (in vitro) samples. (E) Phosphorylation (MFI) of p38MAPK, Akt and ERK in CD14+ monocytes is increased after PMA/ionomycin stimulation compared to isotype controls and ex vivo samples and showed the maximum phosphorylation capacity for each protein. FSC, forward scatter; SSC, side scatter; MFI, median fluorescence intensity. (Data are plotted as the mean ±SEM; n = 5.)

A typical example of monocytes (CD14+) and T-cells (CD3+) gating strategies is depicted in Fig 2A. In line with previous observations, activation of whole blood samples by PMA/ionomycin significantly increased the expression of phosphorylated p38MAPK in CD3+ T-cells compared to the *ex vivo* (unstimulated) whole blood level and isotype controls [37] (Fig 2B and 2C). In CD14+ monocytes, an increase in phosphorylation levels after stimulation was observed for p38MAPK, ERK and Akt compared to the isotype controls and ex vivo samples (Fig 2D and 2E). Again, this increase is in line with previous studies [39–41].

**Fig 3:**
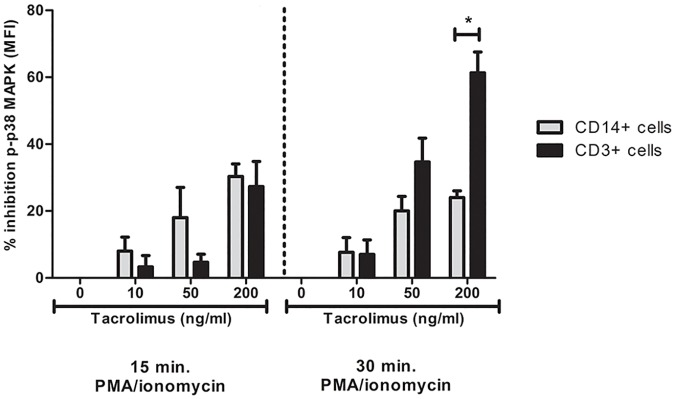
p38MAPK phosphorylation is inhibited more in T-cells than in monocytes. Blood samples from healthy volunteers were spiked with either vehicle, 10 ng/ml tacrolimus, 50 ng/ml tacrolimus or 200 ng/ml tacrolimus. Thereafter, the phosphorylation of p38MAPK was determined in T-cells and monocytes after 15 or 30 min. of stimulation with PMA/ionomycin. After 30 min. stimulation, T-cells were significantly more inhibited than monocytes. (Data are plotted as the mean ±SEM; n = 3) *) p < 0.05; **) p < 0.01; ***) p < 0.001.

To study the effect of tacrolimus on CD3+ T-cells and CD14+ monocytes, cells were incubated with tacrolimus in a dose dependent manner. In this study, T-cell p38MAPK phosphorylation levels served as controls. At a high concentration (200 ng/mL), tacrolimus inhibited p-p38MAPK in T-cells with a mean of 60%, which was significantly higher than the percentage of inhibition in monocytes (30%, p < 0.05) (Fig 3). For the monocytes this was not different over time.

**Fig 4:**
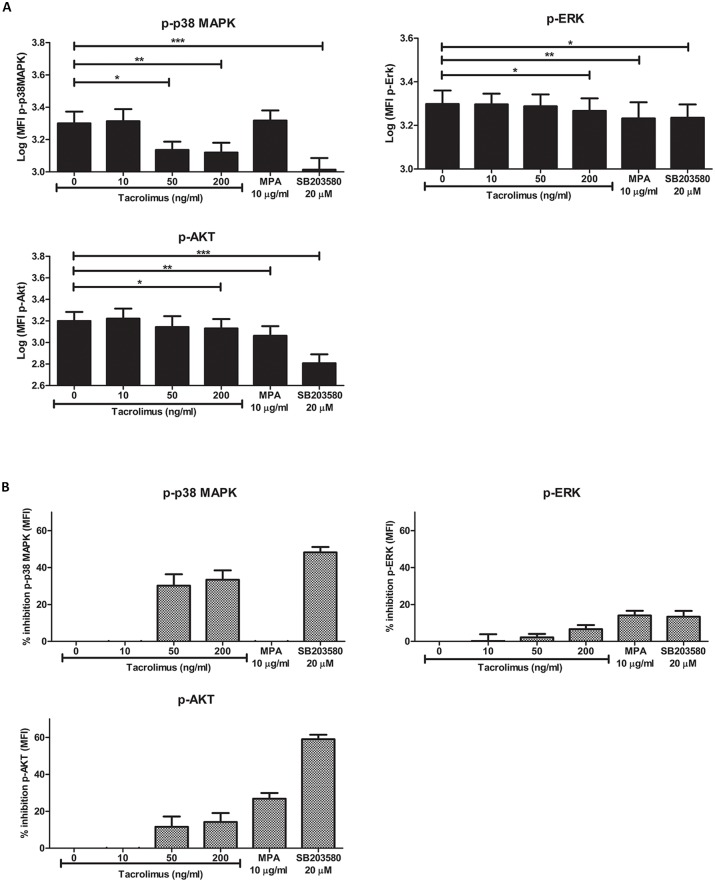
Tacrolimus and MPA can inhibit signaling pathway activation in whole-blood samples. (A) Phospho-p38MAPK (upper left panel), p-ERK (upper right panel) and p-Akt (lower panel) phosphorylation in monocytes was measured as MFI level. Blood samples from healthy volunteers were spiked with vehicle, 10 ng/ml tacrolimus, 50 ng/ml tacrolimus, 200 ng/ml tacrolimus, 10 μg/ml MPA or 20 μM of the MAPK inhibitor SB203580. The effect of tacrolimus and MPA was based on the stimulated samples without the addition of drugs. The MAPK inhibitor was used as a positive control. Gating was performed according to Fig 2. Tacrolimus was found to have an effect on p38MAPK, ERK and Akt phosphorylation. Akt and ERK phosphorylation was decreased in the presence of MPA. (B) Percentages of inhibition for the phosphorylation of p38MAPK (upper left panel), ERK (upper right panel) and Akt (lower panel). Data are normalized for the MFI values of the stimulated samples without the addition of immunosuppressive drugs. (Data are plotted as the mean ±SEM; n = 5) *) p < 0.05; **) p < 0.01; ***) p < 0.001.

Next, the effect of tacrolimus and MPA on the phosphorylation of each signaling protein in CD14+ monocytes was measured and compared to the phosphorylation in the samples without drugs. The p38MAPK inhibitor SB203580, used as a control, showed the maximal inhibitory effect on p38MAPK phosphorylation (inhibition 48%; p < 0.001, Fig 4A and 4B). Apart from the effect on p38MAPK, SB203580 also suppressed the phosphorylation of ERK (mean inhibition 13%; p < 0.05), and Akt (mean inhibition 59%; p < 0.001).

In the presence of high tacrolimus concentrations (50 ng/ml, 200 ng/ml), phospho-p38MAPK was significantly lower expressed (p < 0.05 and p < 0.01, respectively) than in the samples without tacrolimus Fig 4A). This decrease was not seen in the presence of a therapeutic tacrolimus concentration (10 ng/ml). The mean maximal p38MAPK phosphorylation inhibition in monocytes was 30% and 33% at 50 and 200 ng/ml tacrolimus, respectively (Fig 4B). Furthermore, in the presence of 200 ng/ml tacrolimus the PMA/iono activated phosphorylation of both ERK and Akt were suppressed, although with small percentages (mean 7% and 14%, respectively, p < 0.05, Fig 4) On the other hand, MPA (10 μg/ml) downregulated p-ERK and p-AKT (mean inhibition 14% and 27%, respectively, p < 0.01), but not phospho-p38MAPK (p = 0.51, Fig 4).

Taken together, these experiments demonstrate that tacrolimus at high concentrations inhibits phosphorylation of p38MAPK more than Akt and ERK, while at therapeutic concentrations, tacrolimus did not affect the activation of these molecules. In addition, MPA suppresses the phosphorylation of Akt more than the phosphorylation of ERK or p38MAPK.

### IL-1β production decreased in the presence of MPA but not after tacrolimus spiking

**Fig 5:**
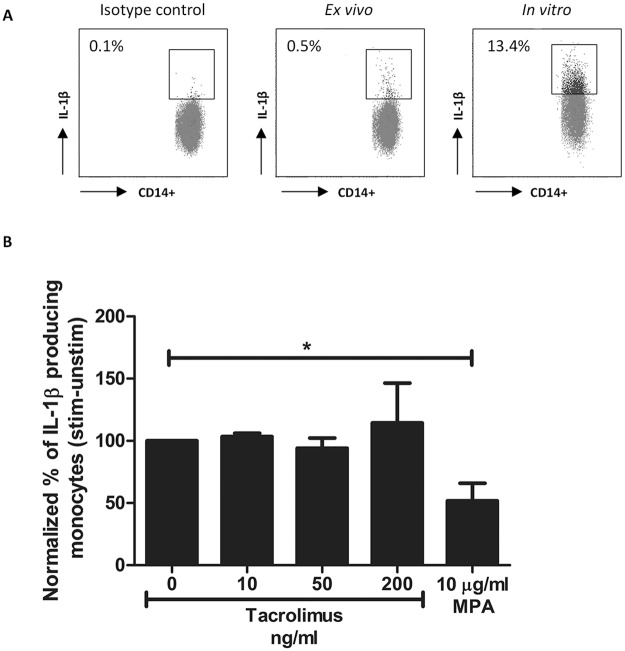
IL-1β production by monocytes of healthy controls is suppressed in the presence of MPA but not in the presence of tacrolimus. (A) Dot plots showing IL-β production with or without stimulation in monocytes. Cells were gated of whole blood samples according to Fig 2a. Isotype controls were used as negative controls and were used to set the gate for the positive IL-1β expression. Results are shown as the percentage of IL-1β producing monocytes compared to the isotype control. Samples were stimulated with PMA/ionomycin for maximum production of IL-1β. (B) Mean percentages of IL-1β producing monocytes after spiking with vehicle, 10 ng/ml tacrolimus, 50 ng/ml tacrolimus or 10 μg/ml MPA. Samples were corrected for the unstimulated results and then normalized to the samples without drug exposure. IL-1β production in monocytes was significantly suppressed by a concentration of 10 μg/ml MPA. (Data are plotted as the mean ±SEM; n = 5) *) p < 0.05.

The percentage of IL-1β-producing cells was studied to determine whether the significant alterations in phosphorylation of the MAPK pathway members p38 and ERK by tacrolimus has an effect on cytokine production by monocytes. Again, whole blood samples were stimulated with PMA/ionomycin after which IL-1β protein expression was measured (Fig 5A). After stimulation, more than 10% of the monocytes expressed the 17.3 kD form of IL-1β, which is the active IL-1β protein (in contrast to the 31kD precursor protein which is not biologically active). No significant change in IL-1β protein expression was found when blood samples were spiked with tacrolimus at either a therapeutic concentration (10 ng/ml, p = 0.28) or a concentration of 50 ng/ml or 200 ng/ml (p = 0.36 and p = 6758, respectively) (Fig 5B). A significantly lower percentage of IL-1β-producing cells was found in the presence of 10 μg/ml MPA (about 50% inhibition; p < 0.05).

### Effects of tacrolimus and MPA on phagocytosis by monocytes

**Fig 6:**
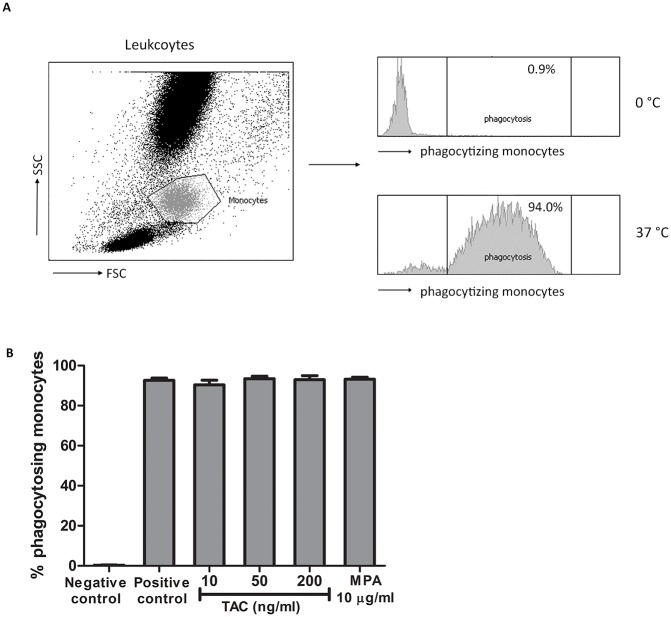
The percentage of phagocytosis by monocytes from healthy controls was not changed in the presence of tacrolimus or MPA. (A) Monocytes were selected from the leukocyte population by a forward and side scatter. Analysis was based on the phagocytosis of the FITC-labeled bacteria. Incubation with FITC-labeled bacteria on 37°C showed a high percentage of phagocytosing monocytes (positive control) compared to monocytes on 0°C (negative control). (B) Mean percentage of phagocytosing monocytes after spiking with either vehicle, 10 ng/ml tacrolimus, 50 ng/ml tacrolimus, 200 ng/ml tacrolimus or 10 μg/ml MPA. Incubation at 37°C increased the percentage of phagocytosing monocytes by more than 90%. Effects of tacrolimus and MPA on phagocytosis were determined as the percentage of phagocytosing monocytes compared to the positive control without immunosuppressive drugs. (Data are plotted as the mean ±SEM; n = 4).

Subsequently, phagocytosis, one of the primary biological functions of monocytes, was studied in the presence and absence of immunosuppressive drugs. The percentage of monocytes that phagocytized labeled bacteria after incubation at 37°C, was more than 90% (Fig 6). This percentage was not influenced by tacrolimus at therapeutic (10 ng/ml, p = 0.44) or high (50 ng/ml, p = 0.29 and 200 ng/ml, p = 0.33) concentrations nor by a high concentration of MPA (10 μg/ml, p = 0.45).

### Phenotypic macrophage differentiation is influenced by tacrolimus and MPA

After stimulation with M-CSF, freshly isolated monocytes differentiate into M1 or M2 subsets when additionally treated with the appropriate triggers (a schematic flow diagram of these experiments is given in S1A Fig). In line with previously published data, M1 macrophages had a higher expression of CD80 and CD64, M2a macrophages had a lower expression of CD14 and a higher expression of CD200R, and M2c macrophages had a higher expression of CD163 and CD16 on their surface compared to macrophages cultured without the addition of cytokines (S1B Fig) [38].

**Fig 7:**
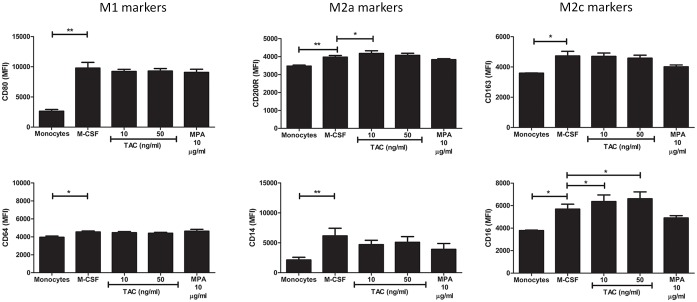
Monocyte differentiation in the presence of tacrolimus and MPA causes a small shift in macrophages subsets. CD14+ monocytes were freshly isolated from whole blood samples of healthy volunteers (n = 5) and then cultured for 4 days with either vehicle, 10 ng/ml tacrolimus, 50 ng/ml tacrolimus, 200 ng/ml tacrolimus or 10 μg/ml MPA. The addition of cytokines was used a positive control. Differentiated macrophages were gated based on their location on the forward sideward scatter. After 4 days of culturing, the expression of all tested surface markers was increased compared to freshly isolated monocytes. The addition of tacrolimus, but not MPA, resulted in an increase of the expression of M2 markers (CD16 and CD200R). (Data are plotted as the mean ±SEM; n = 5 *) p < 0.05; **) p < 0.01; ***) p < 0.001.

Differentiation of monocytes/macrophages can be divided into two processes: maturation of the monocyte into a macrophage and the subsequent polarization of a macrophage into an M1 or M2 type. First, the effect of tacrolimus and MPA on monocyte maturation was studied. After maturation of monocytes under M-CSF culture conditions only, the expression of all six tested markers increased significantly (Fig 7). During maturation, tacrolimus (10 ng/ml), but not MPA, slightly increased the expression of CD200R and CD16 (both markers for M2 macrophages, p < 0.05 Fig 7), compared to the maturation without the presence of immunosuppressive drugs. A high concentration of tacrolimus (50 ng/ml) also increased the expression of CD16 (MFI increase: 5692 to 6607 p < 0.05). Second, the polarization of these mature macrophages was determined. The addition of IFN-γ, IL-4 and IL-10 was used as a positive control for the differentiation assays to compare the polarization of the macrophages after their maturation from monocytes. An increase of CD200R and CD16 expression on monocytes was seen after the addition of IL-4 and IL-10 to the culture medium, which are both stimuli to induce M2 macrophages (S1B Fig). Thus, tacrolimus increased the expression of markers for M2 macrophages, while this was not seen for MPA.

Next, the influence of tacrolimus and MPA on the polarization of macrophage subsets was determined, by measuring the expression of M1- and M2-related surface markers. Monocytes were cultured for 4 days in the presence of M-CSF as a maturation stimulus supplemented with M1 (IFN-γ), M2a (IL-4), and M2c (IL-10) stimulants and different concentrations of tacrolimus or MPA.

**Fig 8:**
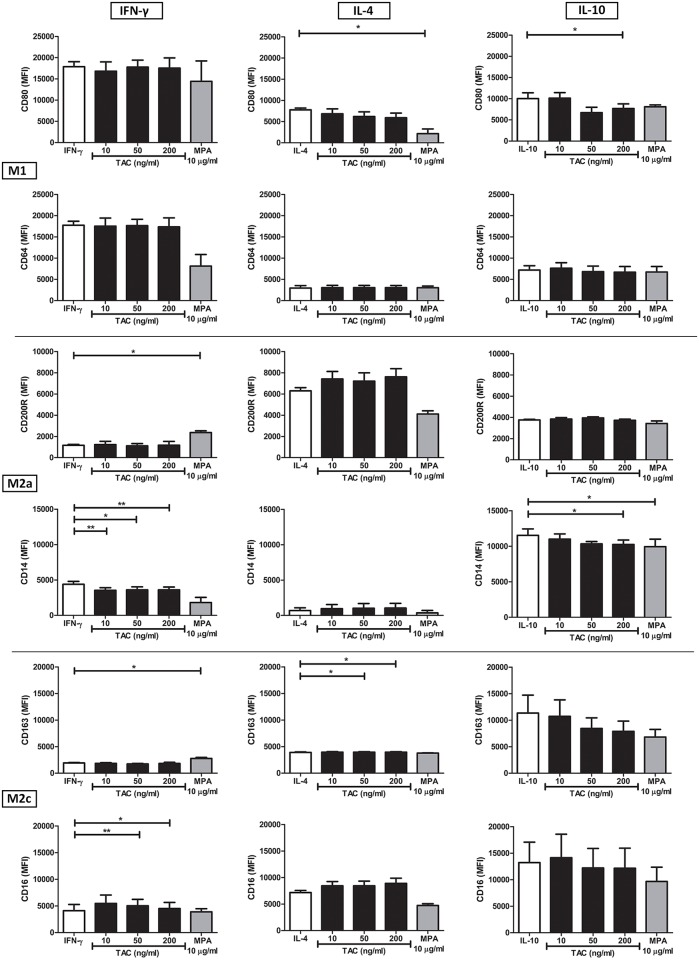
MPA and tacrolimus act differentially on the polarization of M1 and M2 macrophages. CD14+ isolated monocytes were cultured for 4 days in the presence of either M-CSF and IFN-γ (left graphs), M-CSF and IL-4 (middle graphs) or M-CSF and IL-10 (right graphs). In addition, vehicle, 10 ng/ml tacrolimus, 50 ng/ml tacrolimus, 200 ng/ml tacrolimus or 10 μg/ml MPA were added to each culture condition. Tacrolimus changed the expression of M2 markers under a M1-driven condition and decreases the expression of M1 markers under M2 conditions. MPA reduced the expression of CD80 under a M2 inducing condition and increased M2 expression under IFN-γ stimulation. (Data are plotted as the mean ±SEM n = 3 *) p < 0.05; **) p < 0.01; ***) p < 0.001.

In the presence of IFN-γ, a stimulus for M1, the addition of tacrolimus (50 ng/ml and 200 ng/ml) led to the increased expression of the M2 marker CD16 (p < 0.01 and p < 0.05, respectively) and a lower expression of CD14 (p < 0.05 and p < 0.01, respectively, Fig 8, left column). In addition, tacrolimus at a therapeutic concentration (10 ng/ml) also lowered CD14 expression (p < 0.05). MPA caused a significant increase of the other two M2 markers, CD200R and CD163 (p< 0.05) and, although not significant, seemed to lower the expression of the M1 markers CD80 and CD64.

In the presence of the M2a stimulus IL-4, high concentrations of tacrolimus (50 and 200 ng/ml) increased the expression of CD163, although this difference was small (MFI: from 3906 to 3958 and 3957, respectively, p < 0.05, Fig 8, middle column). MPA significantly decreased the expression of CD80 (M1 marker, p < 0.05).

Finally, tacrolimus, but only at a concentration of 200 ng/ml, decreased CD14 and CD80 expression in the presence of IL-10 (M2c stimulus) (p < 0.05), while MPA decreased CD14 expression (p < 0.05) and did not affect the other markers (Fig 8, right column). Thus, both tacrolimus and MPA increased the expression of M2 markers and decreased the expression of M1 markers in the presence of different M1 and M2 stimuli.

## Discussion

Cells from both the adaptive and innate immune system play an important role in the immune response after SOT but the effects of the immunosuppressants tacrolimus and MPA on human monocyte differentiation and functions have not been studied in-depth in previous studies. This study demonstrates that 1) these drugs partially inhibit phosphorylation of signaling molecules involved in CD14+ monocyte activation, i.e., p38MAPK, ERK and Akt kinases, but 2) have only limited effects on cytokine production, phagocytosis, phenotypic maturation, and polarization (an overview of de results is given in S1 Table).

The inhibitory effect of tacrolimus on the calcineurin pathway and the effect of MPA on the reduction of guanosine nucleotide synthesis in T-cells are well-known [19, 42]. Tacrolimus also proved to inhibit p38MAPK phosphorylation in T-cells by more than 60%, which contrasts with the inhibition in monocytes [37]. As demonstrated here, in monocytes the suppression of phosphorylation of intracellular signaling molecules was not more than 35%. Tacrolimus partly inhibited p38 phosphorylation, while MPA mainly inhibited Akt phosphorylation, although this inhibitory effect was also limited. Furthermore, phagocytosis and differentiation were minimally influenced by tacrolimus and MPA, showing that monocytes could be functional in the presence of tacrolimus and MPA. This may imply that cells of the innate immune system are less susceptible for the immunosuppressive effects of tacrolimus or MPA in comparison to cells of the adaptive immune system. The difference in inhibition between the two immune responses could be explained by the working mechanism of the immunosuppressive drugs. For example, the main target of tacrolimus is the calcineurin pathway and it may be that this pathway plays a more important role in T-cell activation than in monocyte activation [43].

The limited effect of tacrolimus on p-ERK, a member of the MAPK pathway, may explain the incomplete inhibition of monocyte activity and function as demonstrated in this study [44]. P-ERK is involved in processes leading to IL-10 production (M2 macrophages), while phosphorylated p38MAPK is essential for IL-12 production, which is in agreement with the observed shift in macrophage polarization [45]. These data are in contradiction with the findings reported by Chang *et al*. who described an inhibitory effect of tacrolimus on p-ERK but not on p38MAPK phosphorylation [26]. However, in contrast to the present study in which primary monocytes were investigated, the experiments by Chang *et al*. were performed with LPS-stimulated monocytic leukemia cells (THP-1) and phosphorylation was measured by means of Western blot. In the present study, phospho-specific flowcytometry was used to quantify the biological effects of tacrolimus and MPA at the single-monocyte level, which is a more sensitive tool for pharmacodynamic monitoring of drug effects [46, 47].

In contrast to tacrolimus, MPA (at a therapeutic concentration of 10 μg/ml) did not suppress the phosphorylation of p38MAPK, affected p-ERK to some degree, and had the largest effect on p-Akt. MPA inhibits inosine monophosphate dehydrogenase, an enzyme that is responsible for the *de novo* synthesis of guanosine nucleotides. As a consequence, the proliferation of B and T-cells is inhibited. The only described effect of MPA on monocyte function is the reduced production of IL-6 and IL-10, which are both downstream products of the Akt/mTOR pathway [42, 48]. It has also been reported that IL-1β is a downstream molecule of the AKT pathway [49, 50]. Here we found that MPA indeed partly inhibited IL-1β production, suggesting that, in combination with the preferred inhibition of p-AKT, MPA affects cytokine production via p-AKT.

The inhibition of signaling molecule phosphorylation by tacrolimus and MPA was smaller than by the positive control SB203580, showing the limited effects of both immunosuppressive drugs. However, the inhibition of phosphorylated p38MAPK by the positive control was also not more than 50%. SB203580 was designed as an inhibitor of the phosphorylation induced by p38MAPK on other molecules and on its own molecule, but cannot inhibit phosphorylation of p38MAPK by other kinases [51]. Here, a significantly inhibitory effect of the MAPK inhibitor on phosphorylated p38MAPK was found, which can probably be ascribed to of auto-phosphorylation in monocytes [52–54]. This alternative, non-canonical pathway for p38MAPK phosphorylation could be another partial explanation for the incomplete inhibition of monocytes by tacrolimus and MPA, besides the unaffected ERK phosphorylation.

The residual phosphorylation of the signaling molecules after tacrolimus or MPA treatment may imply that monocyte functions, such as phagocytosis, remain intact. After SOT, phagocytosis by monocytes/macrophages is one of the mechanisms to overcome infections. Here, phagocytosis of E.coli-bacteria by monocytes was not affected by either tacrolimus or MPA, suggesting that this monocyte function is still active during immunosuppressive drug treatment.

The present study reports for the first time on the change of human macrophage maturation and polarization in the presence of tacrolimus or MPA. Only high concentrations of tacrolimus affect the maturation and change the polarization of macrophages to some extent. Addition of tacrolimus or MPA did not affect the expression of CD80/CD64 (M1). However, tacrolimus did stimulate the expression of CD16 and CD200R (M2). A similar change in polarization by tacrolimus was previously found in mouse studies [55]. In addition, the effect of tacrolimus and MPA on macrophage polarization in the presence of specific stimuli was investigated. A shift to an M2 phenotype was noticed when monocytes were cultured with tacrolimus or MPA in combination with IFN-γ, a cytokine that induces the differentiation of monocytes into M1 macrophages. CD200R and CD163 (M2) expression was increased, although the expression in M1 markers did not change. However, in the presence of M2-driving cytokines (IL-4 or IL-10) the CD80 marker for M1 macrophages was diminished by tacrolimus, again pointing to induction of M2 differentiation. Altogether, these findings suggest that tacrolimus and MPA have a limited effect on the function of monocytes by driving their differentiation towards an M2 phenotype.

In order to reveal the clinical relevance of the limited effect of tacrolimus and MPA on monocytes, the drug effects should be studied in a clinical setting. The role of monocytes in alloreactivity after SOT includes the recognition of non-self antigens during the cellular immune response and danger signals (PAMP’s) induced by ischemia-reperfusion injury [56, 57]. The humoral immune response includes the activation of Fcγ-receptors on monocytes by allo-antibodies [8]. This immune response plays an important role in chronic antibody-mediated rejection which is the main reason for chronic graft loss [58]. The residual monocyte activity, a consequence of the limited effect of tacrolimus and MPA treatment, may partly explain why chronic antibody-mediated rejection occurs after SOT. Furthermore, our previous study on monocytes from kidney transplant patients showed a similarly limited functional effect of immunosuppressive drugs on monocytes, suggesting that these cells can still play a role in early post-transplant cellular immunity [59]. As readout in a new study, signaling protein phosphorylation could be measured, thereby relating the results to the outcomes of the present paper. Here, we focused on the activation and function of CD14+ monocytes, without dividing them into CD16-positive and -negative subsets, which show differential responses in an inflammatory setting [60]. Future studies can focus on these subsets to reveal the effects of immunosuppressive medication on these clinically relevant monocyte subsets. In addition, studies in patients can unveil the effect of combination therapy on monocyte activation and function, since the present study focused on the individual immunosuppressive drug effects. It must also be considered that the role of macrophages may be different in different types of organ transplantation. For example, cardiac macrophages are involved in tissue remodeling and repair after myocardial infarction, while in the lungs their primary role is immune surveillance. In the liver, Kupffer cells are involved in the breakdown of erythrocytes and the response to infections, toxins, ischemia and other stress conditions [61, 62]. These cells are involved in allograft rejection and may also play an important role in the development of immune tolerance after transplantation, suggesting that (partly) inhibition of these cells with tacrolimus and MPA may will cause negative effects on graft survival [62]. In addition, macrophages are known for their heterogeneity and it is possible that the composition of macrophage subsets is different for different organs [63, 64]. For example, the polarization of tissue-resident macrophages is dependent on their local environment, suggesting high heterogeneity in macrophage subsets between different organs [65]. Research is needed to show the functional effects of tacrolimus and MPA on these cells. However, each macrophage does have targets for tacrolimus and MPA (e.g. calcineurin pathway and inosine monophosphate dehydrogenase) suggesting that the difference in sensibility for these drugs between different macrophage subsets at the single cell level may be limited.

In conclusion, tacrolimus and MPA hardly suppress monocyte signaling pathway activation. The residual phosphorylation of signaling proteins explains the limited effect of both immunosuppressive drugs on cytokine production and phagocytosis, apart from monocyte differentiation. This suggests that innate immune responses induced by monocytes after SOT may still occur despite immunosuppressive therapy.

## Supporting Information

S1 Fig Monocyte differentiation experiments. (A) Schematic overview of the monocyte differentiation experiments. For the first part of the differentiation study, monocytes were cultured in the presence of M-SCF to induce maturation into macrophages. Next, the expression of the surface markers for M1, M2a or M2c macrophages was determined after addition of tacrolimus or MPA to the culture system. In the second part of the experiments, monocytes were induced to polarize into a specific macrophage subtype. Addition of IFN-γ drives the monocytes to polarize into M1 macrophages, IL-4 induces M2a and IL-10 increases M2c macrophages. Then, tacrolimus and MPA were added to the cultured cells to determine the capability of both drugs to change the expression of surface markers on M1, M2a and M2c differentiated macrophages. (B) Validation of the differentiation assay. After addition of IFN-γ to the culture medium, monocytes are stimulated to differentiate into M1 macrophages with a significantly higher expression of the CD80 (p < 0.001) and CD64 (p < 0.001) compared to monocytes cultured without the addition of extra cytokines. Culturing with IL-4 increased the expression of CD200R (p < 0.01), lowered CD14 expression (p < 0.01), and thus drove the differentiation into M2a macrophages. IL-10 stimulation induced the expression of CD163 (p < 0.01) and CD16 (p < 0.05) and resulted in M2c macrophage differentiation. (Data are plotted as the mean ±SEM; n = 5) *) p < 0.05; **) p < 0.01; ***) p < 0.001.
(TIF)

S1 Table Overview of the effects of tacrolimus and MPA on monocyte activation and function. (PDF)
