# Supplementary figures and images for: The Effect of Tacrolimus and Mycophenolic Acid on CD14+ Monocyte Activation and Function
Source: PLoS One. 2017 Jan 25;12(1):e0170806. doi: 10.1371/journal.pone.0170806 (PMC5266297; doi:10.1371/journal.pone.0170806)

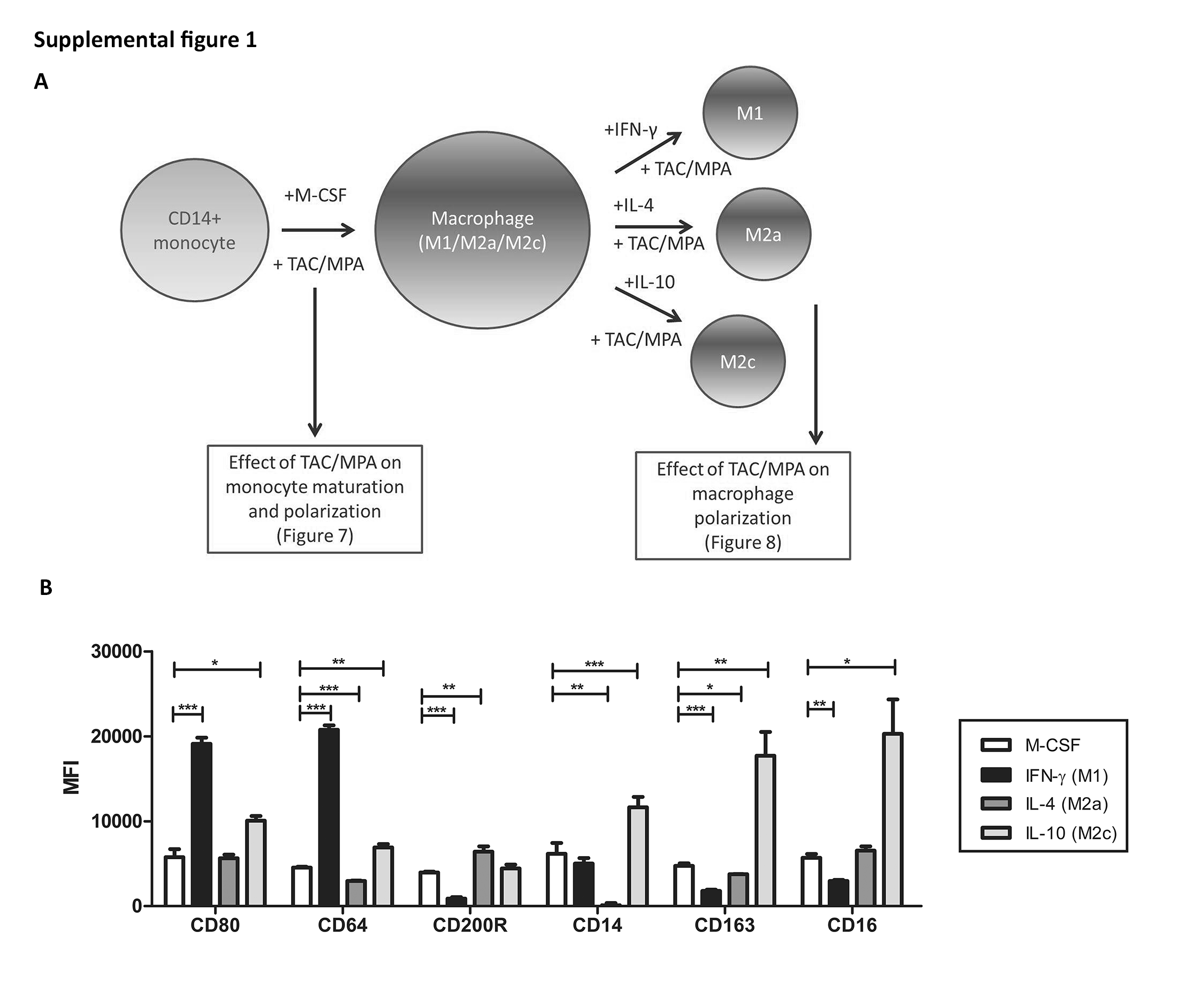

Supplement: S1 Fig — (A) Schematic overview of the monocyte differentiation experiments. For the first part of the differentiation study, monocytes were cultured in the presence of M-SCF to induce maturation into macrophages. Next, the expression of the surface markers for M1, M2a or M2c macrophages was determined after addition of tacrolimus or MPA to the culture system. In the second part of the experiments, monocytes were induced to polarize into a specific macrophage subtype. Addition of IFN-γ drives the monocytes to polarize into M1 macrophages, IL-4 induces M2a and IL-10 increases M2c macrophages. Then, tacrolimus and MPA were added to the cultured cells to determine the capability of both drugs to change the expression of surface markers on M1, M2a and M2c differentiated macrophages. (B) Validation of the differentiation assay. After addition of IFN-γ to the culture medium, monocytes are stimulated to differentiate into M1 macrophages with a significantly higher expression of the CD80 (p < 0.001) and CD64 (p < 0.001) compared to monocytes cultured without the addition of extra cytokines. Culturing with IL-4 increased the expression of CD200R (p < 0.01), lowered CD14 expression (p < 0.01), and thus drove the differentiation into M2a macrophages. IL-10 stimulation induced the expression of CD163 (p < 0.01) and CD16 (p < 0.05) and resulted in M2c macrophage differentiation. (Data are plotted as the mean ±SEM; n = 5) *) p < 0.05; **) p < 0.01; ***) p < 0.001. (TIF) [file pone.0170806.s001.tif]
