# Supplementary material for: The Effect of Tacrolimus and Mycophenolic Acid on CD14+ Monocyte Activation and Function
Source: PLoS One. 2017 Jan 25;12(1):e0170806. doi: 10.1371/journal.pone.0170806 (PMC5266297; doi:10.1371/journal.pone.0170806)
Supplement: S1 Table — (PDF) [file pone.0170806.s002.pdf]

S2 Table. Overview of the effects of tacrolimus and MPA on monocyte activation and function

	Tacrolimus	MPA	Comments
**Signaling pathways**	+	+	Maximum of 30%
**Cytokine production**	-	+	50% inhibition by MPA
**Phagocytosis**	-	-	
**Differentiation**	+/-	+/-	Change in M2 expression markers, but not all subset markers

+) effect; +/-) small effect; -) no effect
